# Widening community participation in preparing for climate-related disasters in Japan

**DOI:** 10.14324/111.444/ucloe.000053

**Published:** 2022-12-23

**Authors:** Kaori Kitagawa, Subhajyoti Samaddar

**Affiliations:** 1 IOE (Institute of Education), UCL’s Faculty of Education and Society (University College London, UK), 20 Bedford Way, London WC1H 0AH, UK; 2 Disaster Prevention Research Institute, Kyoto University, Gokasho, Uji City, Kyoto Prefecture, 611-0011, Japan

**Keywords:** participation, participatory approaches, widening participation, community-based DRR, climate-related disaster, EAST framework, behavioural insights

## Abstract

This paper discusses community participation drawing on ongoing disaster recovery and preparedness projects (RPP) in the communities affected by the Heavy Rain Event of 2018 in western Japan. Participatory approaches have become a mainstream methodology for community-based disaster risk reduction (DRR) as advocated in the Sendai Framework for Disaster Risk Reduction 2015–2030. The majority of participation research addresses either ‘success’ factors for participation or the types of participation. The paper proposes a notion of ‘widening participation’ in addressing the challenge of attracting people to participate in preparedness initiatives. Originally widening participation was a higher education policy in the UK aiming to broaden the demographic composition of the student base. Even the RPP that are publicly recognised as ‘good practices’ struggle to recruit more people for the projects. Borrowing the notion of widening participation, the paper identifies how each project encourages non-participants to get involved in the project activities. The paper applies the EAST framework (Easy, Attractive, Social, Timely) widely utilised in the policy making of widening participation and further public services. Rather than providing the public with information and guidance, ‘easy’, ‘attractive’, ‘social’ and ‘timely’ behavioural approaches tend to enable participation. Examining these four principles in the four cases of RPP, the paper suggests that the EAST framework is feasible in strengthening the strategies for widening participation in preparedness action. The paper, however, recognises a need to address the difference between top-down public policies and bottom-up community projects in the application of the framework.

## Introduction

This paper discusses community participation drawing on ongoing disaster recovery and preparedness projects (RPP) in the communities affected by the Heavy Rain Event of July 2018 centred on west Japan. ‘Preparedness’ action is considered to be undertaken as part of disaster risk reduction (DRR) measures to build capacities for effective response and smooth recovery against all types of disasters [1]. The empirical studies carried out in Okayama and Ehime Prefectures captured the transition from recovery to preparedness following the Heavy Rain Event. The paper depicts the situations of community participation in each RPP in the transition. For ‘community’, the paper refers to ‘a community of interest’ formed in a certain geographical location by local residents. There is a rich body of literature available on participation, most of which addresses either ‘success’ factors for participation or the types of participation. The paper proposes ‘widening participation’ is another dimension in considering participation, particularly for those ongoing initiatives that are working well. Originally ‘widening participation’ was a higher education (HE) policy in the UK introduced in the 1990s to broaden the demographic composition of the student base [2]. By removing barriers that disadvantaged groups of young people might face, widening participation policy aims to improve their access to education and progression in HE to raise their life chances. Hence, ‘widening’ addresses not only the increase in the number of students in HE but diversifies the composition of the student demographics. This paper explores an application of this notion in examining participatory approaches to building preparedness in communities.

‘Community-based’ and ‘participatory’ approaches have become a mainstream methodology for ‘all of society engagement’ in DRR as advocated in the Sendai Framework for Disaster Risk Reduction 2015–2030 [3]. In 1989, Maskrey argued for the significance of community engagement in disaster mitigation schemes [4]. Since many donors and researchers have developed and implemented a range of innovative participatory programmes, together with local communities, organisations and authorities (e.g., Kitagawa, 2019) [5–9]. Based on the perspective that ‘CBDRM [community-based disaster risk management] is a participatory process’ [9], ‘participatory DRR’ has often been used as a synonym for community-based DRR. The paper focuses on ‘participation’ but uses ‘community-based’ in referring to the studied cases in differentiating them from top-down and authority-led projects.

Japan is one of the disaster-prone countries that has keenly promoted community-based DRR. The 2013 revised Disaster Countermeasures Basic Act introduced a new system of Community Disaster Management Plans [10]. Every community is encouraged to create a plan. Japan has a policy framework of public help [*kojo*], self-help [*jijo*] and collaborative help [*kyojo*], which emphasises the importance of balancing the three. Since the devastating experience of the 2011 Great East Japan Earthquake and Tsunami, policymakers and experts have stressed the tripartite framework even more in preparing for forthcoming disasters [11,12]. One of the key measures has been the system of Community Disaster Management Plans. Aiming for a collaborative model for DRR, the system enables community residents to participate in the process of developing a plan, together with the municipal government disaster management council. The government has offered subsidies and expertise to support communities in creating plans [10]. We also touch upon a relevant policy term in Japan: *machizukuri* [community development]. It is an overarching social policy integrating health, welfare, education and DRR, which was often mentioned in our empirical study. Participation is emphasised as a requisite of *machizukuri* in developing a resilient and sustainable community.

The paper argues that widening participation is one of the common challenges in participatory and community-based DRR, particularly in ongoing projects, and requires strategic planning. Involving more people in decision-making is critical in building a democratic society. The EAST principles (Easy, Attractive, Social, Timely) have potential as a guidance or evaluation framework for effective widening participation strategies in DRR. The paper first describes the Heavy Rain Event of 2018 and its major damages in the affected areas. This is followed by an explanation of the methodology of the study. The empirical investigation was conducted in October and November 2021. The next section reviews participation literature and discusses the background and discourse of widening participation. The paper then sets up the EAST framework as a conceptual framework for the subsequent analysis. The findings section describes the four RPPs identifying their widening participation strategies applying the EAST principles. The discussion and conclusion further consider the applicability and limitation of the EAST framework in participatory DRR.

## The Heavy Rain Event of July 2018 in western Japan and its impacts

**Figure 1:**
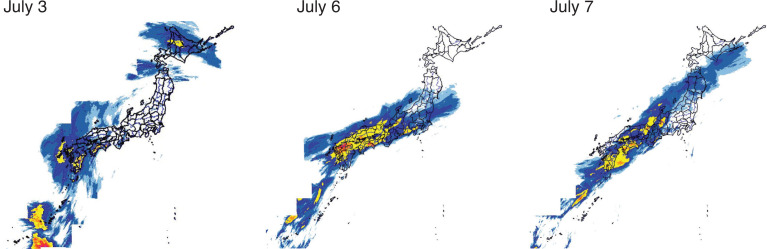
Daily Precipitation (1-km-mesh precipitation distribution obtained by analysing data from weather radars, AMeDAS, and other rain gauge systems). © 2017 Disaster Management, Cabinet Office.

**Figure 2:**
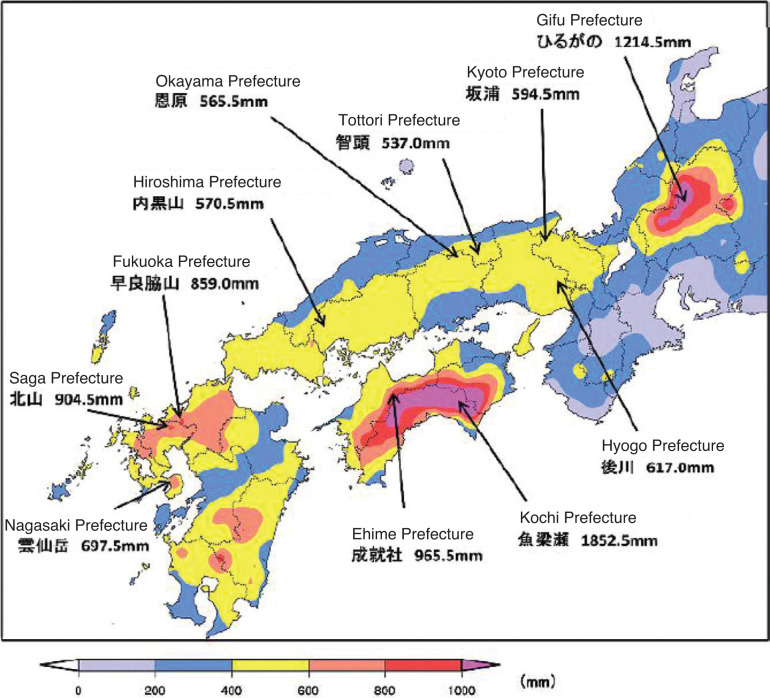
Precipitation distribution during the event (between 00:00 on 28th June and 24:00 on 8th July). © 2017 Disaster Management, Cabinet Office.

**Figure 3:**
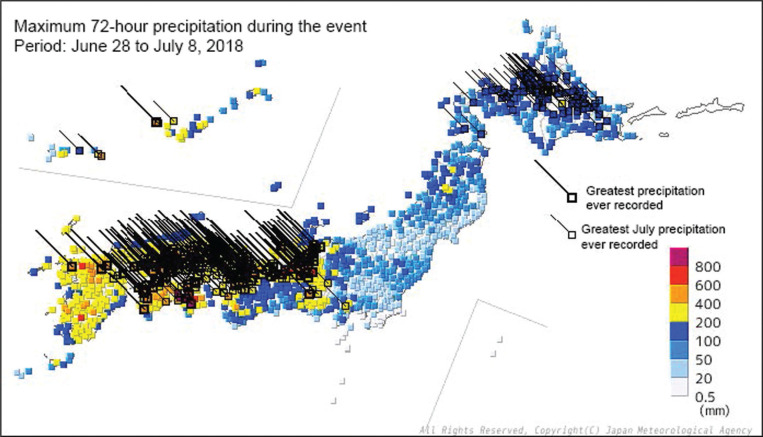
Distribution of the maximum 72-hour precipitation during the event. © 2017 Disaster Management, Cabinet Office.

**Figure 4:**
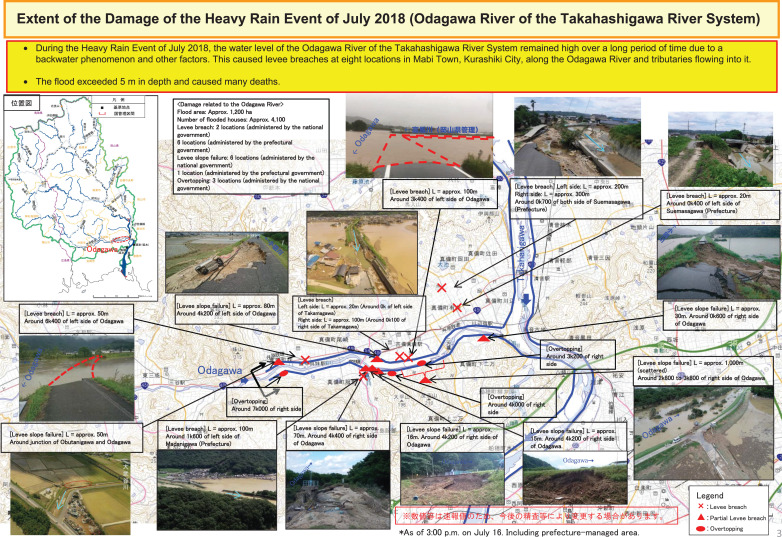
Levee breaches in the Takahashi River, Kurashiki City, Okayama Prefecture. © 2017 Disaster Management, Cabinet Office.

On the 5 July 2018, the rain front halted in western Japan, and the following 3 days, forming 15 line-shaped precipitation systems. Coupled with Typhoon Prapiroon, which emerged around Okinawa Island on the 2 July, the rain front brought record-breaking rains across Japan, particularly in western Japan. The total precipitation exceeded 1800 mm from the 28 June to the 8 July in Shikoku Island and 1200 mm in the Tokai region – two to four times more than the average monthly precipitation in July. The largest 24-hour, 48-hour and 72-hour precipitations in recorded history were observed in the northern Kyushu, Shikoku, Chugoku, Kinki, Tokai and Hokkaido regions (Figs 1–4) [13]. The Japan Meteorological Agency Advisory Panel suggested the Heavy Rain Event could be linked to climate change, associated with a long-term trend of temperature increase and a similar increasing trend in the amount of water vapour in the air [14].

Causing river flooding, inundation and sediment, the Heavy Rain Event left serious damage in these regions: 237 persons dead, eight missing and 432 injured as shown in Table 1. In the event, 6767 houses were completely destroyed, 15,234 half or partially damaged and 28,469 inundated as indicated in Table 2 [13].

**Table 1.** Human casualties (as of 9 January 2019)

Prefecture	Fatality	Missing persons	Seriously injured	Lightly injured
Okayama	66	3	9	152
Hiroshima	115	5	61	85
Ehime	31		33	2
Others	25		20	70
Total	237	8	123	309

**Table 2.** Houses damaged (as of 9 January 2019)

Prefecture	Completely destroyed	Half-destroyed	Partially damaged	Above-floor flooding	Below-floor flooding
Okayama	4828	3302	1131	1666	446
Hiroshima	1150	3602	2119	3158	5,799
Ehime	625	3108	207	187	2492
Others	164	1,231	534	2,162	7559
Total	6767	112,433	3991	7173	21,296

In terms of infrastructure, power outages affected approximately 80,000 households, gas disruption affected approximately 290 households and water outages occurred in 80 municipalities in 18 prefectures, affecting approximately 260,000 households. Power and gas supplies were recovered on the 13 July and 8 July, respectively, while water supply was not restored until 13 August [13].

As a result of these circumstances, 28,000 evacuees materialised (2500 in the Okayama Prefecture, 800 in the Ehime Prefecture), most of whom stayed in shelters, which eventually numbered 3779 (436 in the Okayama Prefecture, 462 in the Ehime Prefecture). One month after the disaster, the evacuee number dropped to 3500, and all shelters, except for some welfare shelters, were closed in December [13].

The major damage in the four field sites is summarised in Table 2 and some images can be found in Table 3 [18–20].

**Table 3.** Damage of the 2018 disaster in field sites

Damage	Kawabe Ward, Mabi Town, Kurashiki City, Okayama Pref	Yata Ward, Mabi Town, Kurashiki City, Okayama Pref	Miyoshi Ward, Ozu City, Ehime Pref	Nomura Town, Seiyo City, Ehime Pref
**Population/households**	4394/1734 as of 30 June 2018	4951/1970 as of 30 June 2018	885/403 as of 30 June 2018	3424/8359 as of 1 October 2015
**Completely/partly destroyed occupied houses**	4646/846 Kurashiki City as a whole as of 5 April 2019		1/57	117/215
**Dead/injured persons**	52/120 Kurashiki City as a whole as of 5 April 2019		0/0	5/0

Source: Mabi Town [15]; Miyoshi Ward data provided by Miyoshi Community Centre; Nomura Town [16,17].

## Methodology

This study is of an interpretivist nature and employs a case study approach investigating four community-based participatory RPPs in the western part of Japan. Three years on since the disaster, these communities have started engaging in preparedness activities moving forward from the recovery phase. Those projects comprise various preparedness activities through which local residents experience participation. The study was guided by the following research question: *What strategies do communities use to widen community participation in RPPs?*

The study set the following criteria for the selection of the case projects:

1. The project is self-managed by the community of interest, which engages in the planning, implementing and evaluating of preparedness activities;
2. Those activities are participatory and collaborative;
3. The project involves collaboration with various stakeholders, such as local governments, non-profit organisations (NPO)/non-governmental organisations (NGOs) and expert academics in the field of DRR;
4. The project aims to reduce the disaster risk of future torrential rain and flooding;
5. The project participants have learned/are learning from their previous disaster experiences that have led to their preparedness action.

Four projects were identified by two means. Two projects were selected from the awardees of the Disaster Prevention and Community Building Awards [21] administered by the Japan Fire and Disaster Prevention Association, which was available in the public domain. The other two projects were introduced via our academic networks. Two researchers invited us to their fields in which they undertake recovery and community development [*machizukuri*] together with local residents.

The starting point of the study was these four projects, which were regarded as ‘good practices’. The first two projects in particular have had much media exposure [22,23]. Three have received recognition and awards from the Japanese government and agencies. In 2020 the Aruku Project was awarded the Regional Revitalization Award organised by 46 regional newspapers and Kyodo News [23,24]. The Satsuki Project recently received the Prime Minister’s Award for the Distinguished Persons in the Contribution to DRR [25]. The project was also one of the awardees of the 25th Disaster Prevention and Community Building Awards administered by the Japan Fire and Disaster Prevention Association [21]. The Evacuation Card Project received the same award the previous year. The fourth project has not yet been officially recognised given it is still at an early stage as a larger-scale scheme. Anecdotally, however, stakeholders including the researcher who is one of the main architects of the recovery project have confirmed its firm implementation process and the community’s high level of social capital. Our observation of their engagement also suggested their commitment and solidarity. We are therefore tentatively including the project as one of the cases.

The main methods of data collection were semi-structured interviews and site observations. In total, 17 stakeholders were interviewed. This comprised nine females and eight males, aged between their 30s and their 70s. These methods allow the gathering of real-life narratives, which enabled us to grasp historical experiences and socio-cultural perceptions of the communities around disaster, community and participation. As a pilot study, the sample size was small. But it served the purpose of discussing each project’s approach to promoting participation. With the interviewees’ permission, the interviews were recorded and summarised. The interviewees are anonymised and quoted using the coded references indicated in Table 4.

**Table 4.** Details of the four case projects

Project title	Aruku Project (A)	Satsuki Project (B)	Evacuation Card Project (C)	Nomura Recovery Project (D)
**Location**	Kawabe Ward, Mabi Town, Kurashiki City, Okayama Prefecture	Yata Ward, Mabi Town, Kurashiki City	Miyoshi Ward, Ozu City, Ehime Prefecture	Nomura Town, Seiyo City, Ehime Prefecture
**Disaster experience**	Floods from rivers	Floods from rivers	Flood from inland waters, floods from rivers	Typhoons, floods from inland waters, earthquakes
**Interviewees**	Leader (A-L)* 3 project members (A-M1, -M2, -M3) 1 researcher (AB-R) 1 NGO (AB-N) 1 local government official (AB-O)	Leader (B-L) 1 project member (B-M) 1 researcher (AB-R) 1 NGO (AB-N) 1 local government official (AB-O)	Leader (C-L) 2 project members and 2 residents (C-M1, -M2, -M3, M4) 1 local government official (C-O) (Group interview)	Leader (D-LO) 2 researchers (D-R1, -R2) 1 local government official (D-LO)
**Observations**	Primary school’s DRR walk in the ward	Exercise and teatime for the residents	–	Primary school’s DRR education class; high school’s group discussion

*L – leader; M – member; R – researcher; N – NGO; O – government official.

## Introducing a widening participation agenda to participatory disaster risk reduction

There is a growing number of DRR studies that question the treatment of participatory approaches as a panacea [26–28]. Evidence is available that communities remained vulnerable to risks even after the ‘successful’ completion of the participatory projects, that external partners’ interests were prioritised over communities’ needs and that the quality of participatory action was poor [29–31]. One of the reasons behind these inadequate outcomes is considered to be a lack of a comprehensive structure that allows researchers and providers to systematically evaluate the effectiveness of participatory approaches [32,33]. Two major streams of participation research emerged. The first derived from other disciplines defining participation as dependent on two factors: process-based criteria and outcome-based criteria [34–36]. The process entails the quality of the participation, such as fairness, accountability and transparency, and researchers deploy different sets of variables. However, an effective process does not necessarily yield the desired results as participation is context-specific and time-dependent, and from there, outcome-based criteria, such as ownership, self-reliance and sustainability were proposed [34,37]. The other stream of participation research applies the typology-based definition of participation, which argues that participation is stratified based on the quality and intensity of engagement [33,35,38]. Participants at the bottom of the participatory ladder are viewed as passive recipients of information, while those at the top are seen as active problem solvers being involved in democratic processes [39].

The above participatory structures address either of these two themes – ‘success’ factors or types of participation [40]. We propose a discussion on the challenge of expanding participants, particularly in already functioning initiatives. Such perspective is pertinent in countries such as Japan, where participatory and community-based approaches to DRR have been widespread. The government and interest groups have created awards to celebrate achievements and promote more engagement. Accumulated knowledge and experience are shared via government websites, media programmes, as well as in academic publications. In many cases, however, some proactive enthusiasts lead DRR initiatives, while those with less interest in DRR remain uninvolved. How to engage the latter group of people tends to be a common challenge in many communities.

This paper borrows the notion of ‘widening participation’ from HE, which provides us with a direction as to how existing initiatives could grow further. Originally, widening participation refers to an approach which addresses ‘discrepancies in the take-up of higher education opportunities between different under-represented groups of students in the UK context’ [2]. One of the underlying aims of such an approach is social cohesion. By removing barriers that non-traditional groups of young people – mainly working-class and ethnic minority students – might face, widening participation policy aims to improve their access to and progression in higher education to enable social mobility. The UK government identifies four drivers of social mobility: ‘conditions of childhood’, ‘educational opportunities and quality of schooling’, ‘work opportunities for young people’ and ‘social capital and connections’ [41]. Providing ‘opportunities for access to higher education’, which falls under ‘educational opportunities and quality of schooling’, has been a flagship policy to raise young people’s life chances. The schemes such as ‘the removal of student number controls, increased availability of bursaries and support and targeted information campaigns’ [42] have been implemented to enable widening participation across the country. As a result, enrolment in higher education has become the highest in its history [41]. The new ‘inclusive’ system ‘has broken the cycle of social reproduction and social inequality within the under-represented groups who were historically excluded from HE’ [43].

At the same time, complex analyses have been reported. For example, there is a clear geographical divide. Less than half of young students from the most disadvantaged areas in England entered higher education compared to those from the most advantaged areas [44]. Moreover, the students from the former group are less likely to apply to high-ranking universities than those of their middle-class counterparts, even though financial and other forms of support are available from such universities to encourage non-traditional students to apply [42]. This trend is referred to as ‘increased stratification’ [45] – a new divide between the universities for non-traditional students and those for traditional counterparts. There is evidence to suggest providing target students with a range of information about HE such as employment options and economic benefits does raise their awareness but does not necessarily lead them to take an action to apply [46]. Recognising this gap in widening participation policy, HE experts have developed tools for more effective approaches, one of them being the EAST framework.

## The EAST framework as an analytical reference

The EAST framework was proposed by Hume and Shelley [42] in the Behavioural Insights Team at the Higher Education Policy Institute. According to the Organisation for Economic Co-operation and Development (OECD) [47], ‘behavioural insights’ refer to:

*an inductive approach to policy making that combines insights from psychology, cognitive science, and social science with empirically tested results to discover how humans actually make choices.*

Behavioural insights aim to inform policy decisions by understanding how people make choices and change behaviour. The approach has been widely used in public services allowing low cost interventions for better service outcomes [48]. Currently, 202 institutions across the world utilise behavioural insights in public policy making [47].

The EAST framework comprises four basic principles, ‘easy’, ‘attractive’, ‘social’ and ‘timely’, which inform the development of effective policies or initiatives in prompting people to take action for better life choices [49]. Some of the most relevant elements of these principles are as follows. ‘Easy’ stands for breaking down a complex goal into simpler and easier actions. People are more encouraged to get involved when the message is straightforward. For ‘attractive’, many of us tend to make an action when our attention and interest are caught. Financial incentives are a clear example, but also beautiful images or bright colours could be effective in inviting people to get involved. ‘Social’ connections drive people to engage in something that they would not if they were on their own. Positive collective action and mutual support can be prompted through interactions and relationships. ‘Timely’ is about narrowing the ‘gap between intentions and actual behaviour’ [49]. People are likely to do something when they are most receptive. The EAST framework has been applied in various policy settings, widening participation in HE being one of them. One of the examples that Hume and Shelley [42] refer to is the use of ‘a relatable role model’, who gives a talk to a disadvantaged group of young people as to how s/he benefited from going to a university. The role model makes the talk ‘attractive’ by sharing the positive gains such as confidence, awareness and employment and also ‘social’ by linking her/his background with the audience’s reality. It is intended that the students will be able to relate themselves to the route into HE and decide to apply.

This paper explores an application of the EAST framework in community participation in RPPs. Despite being two different social agendas, how widening participation in increasing social mobility has been promoted in HE resonates with how community participation in building disaster resilience has been stressed in DRR. Since ‘community-based’ approaches were advocated in the Hyogo Framework for Action, which was updated in the Sendai Framework for DRR as ‘all of the society engagement’, experts and policy makers have put the general public’s participation at the heart of DRR policy. A large number of research papers and project reports on participatory approaches are evidence that they have become the mainstream methodology in DRR. Despite the positive outcome of the expansion in participatory approaches, there are findings to demonstrate that unresolved challenges remain. One of them is the limitation to the number of participants, to which this paper pays attention.

Regardless of the positive recognition that the four projects have received, our empirical study identified their common challenge. The RPPs have been led by strong leadership and supported by enthusiastic members. They certainly own their projects exercising agency. Beyond this core group, however, each project admits a struggle in expanding the number of participants. Here are testimonies. For the Aruku Project, at one of the seminar series we observed, the chairman of the Kawabe Ward Machizukuri Committee made the following comment: ‘Kawabe is doing great. But always by the same members. We need to involve a wider population’. This was echoed by the researcher: ‘the people represented in Aruku are highly motivated and engaged in DRR and *machizukuri*. How to broaden the participation is the challenge’ (AB-R interview). Referring to both the Aruku Project and the Satsuki Project, the NGO staff member analyses that ‘it is difficult to include a wider audience. The rest of the residents don’t get involved, not interested in DRR. How to engage indifferent people in DRR is always difficult, wherever it is. We tried to have a booth in a shopping mall. We also try to involve kids in DRR’ (AB-N interview). In the Miyoshi Ward, the leader reflected, ‘at the time of the Evacuation Card Project, there were reluctant people’ who were not keen on getting involved in creating the card (C-L interview). The researcher observed that ‘I am only seeing “strong” parts of the Nomura community. I am aware I am still missing those who aren’t involved’ (D-R1 interview). This was confirmed by another interviewee – in reference to the ongoing DRR seminar series, ‘The majority is not participating! Those who participate are 30–100 out of 4,000’, for example, in a seminar series. ‘I am not necessarily trying to encourage more people to participate. It takes energy to persuade people to participate’ (D-LO interview). These testimonies commonly show the struggle of the projects to attract a wider audience and achieve a larger number of local residents to get involved in the projects despite demonstrating good practices. Motivating more residents to participate is significant in the realising of a democratic society. This common challenge is worth investigating in deepening the discussion on participation.

## Four recovery and preparedness projects and widening participation strategies

This section first describes the development and aims of each RPP and then examines its widening participation strategy focusing on its key initiative with a reference to the EAST framework. To note, the ‘timely’ aspect is considered to be applicable to all initiatives given they were a response to the flood disaster when people were likely to be amenable.

### The Aruku Project in Kawabe Ward, Mabi Town, Kurashiki City, Okayama Prefecture

#### Background and aims

The full title of the project is the Kawabe Recovery Project Aruku established in the Kawabe Ward, which had 99% of its households inundated above floor level by the 2018 flooding. The Aruku Project aims to develop activities which 1) develop connections and friendships amongst residents and also their passion for life [*ikigai*], 2) create a safe community, 3) retain the disaster experience and prevent it from weathering. The project now has a permanent office offering social spaces for the people of Kawabe [50]. Starting with 20 ‘friends’ in Group LINE (a social media platform similar to WhatsApp widely used in Japan), the number has now gone up to 600. Municipal officials are included as well (A-L interview).

The project was begun by one person shortly after the flooding occurred on 9 July 2018. She started Group LINE with 20 friends asking what they needed. She wanted to reach out to those who were facing difficulties. Her situation was better as she had been able to evacuate to her parents living next to Kawabe and all essentials were available to her family. LINE became a platform to exchange vital information such as where temporary toilets were available in Kawabe. ‘Friends’ rose to 100 that evening. The friends started to mourn that they would not return to where they had lived. Kawabe did not have a support hub. There was little motivation for recovery. Residents had to travel to receive a donation. So she started a food bank in Kawabe involving a local MP. At the end of August, 300 people queued for the food bank. Interestingly, queuing often started 2 hours before the food was served. People wanted to get together and have a chat. Kids were playing cards. ‘Space’ was needed. By September, the number of LINE friends became 300, and the Aruku Project was launched in October with 20 volunteers who wanted to lend a hand (A-L interview).

The shift of getting involved in preparedness occurred a year after the disaster. According to a member of the NGO, ‘the driving force for preparedness was Aruku members regret [of] not being able to help neighbours who passed away in the 2018 event. One year on, they strongly felt they were obliged not to repeat the same mistake’ (AB-N interview). The people in Kawabe were also showing a rising awareness of preparedness through the LINE surveys the project undertook. The survey response during the first six months after the disaster was dominated by demands requesting help, but the contents gradually included forward-looking and proactive comments on DRR (A-L interview). The Aruku Project is now focusing on preparedness action. In fact, at the time of our visit, the members were considering removing ‘recovery’ from the project title given they have been engaged more in preparedness lately (A-M1 interview). Responding to the survey results, the project has introduced a range of preparedness activities including informal classes and seminar series.

#### Yellow Ribbon – ‘easy’ and ‘attractive’

Along with the above preparedness initiatives, the Aruku Project introduced an emergency safety check, Yellow Ribbon [*Kiiroi tasuki*] [51]. The activity is to tie the ribbon at the entrance of your property to show you and your family are safe. No ribbon indicates someone has to call out for [*koe kake*] the residents inside and offer support.

The idea for this initiative stems from a painful lesson learned by many people in Kawabe. They were unprepared, not being able to call out enough or taking too long to reach out to those in need of help during the 2018 disaster. As the leader explains,

*Kawabe didn’t have an evacuation centre, nowhere to evacuate – residents were relying on top-down instruction and hard measures* (A-L interview).

Nevertheless, the top-down instruction did not really work.

*Official risk communication covers a wide area. The authority’s area mail ‘the river was collapsed’ was not helpful at all because it didn’t tell us what I should do. We didn’t understand the information so couldn’t link it to our own behaviour* (A-L interview).

Their realisation was that:

*The trigger might be a top-down instruction, but what to do next is up to us. We need to think about how to save our lives. It is our responsibility to question and make changes* (A-L interview).

The Aruku Project, therefore, developed ‘personalised’ (A-L interview) preparedness initiatives, Yellow Ribbon being one of them. The Kawabe Ward had 1700 households before the disaster, which decreased to 1500, of which 1300 had a yellow ribbon distributed by March 2021. In May, the project organised a safety-check drill in which 65.8% out of 1300 took part. The researcher supporting the Aruku Project clearly indicated that:

*To get people involved and have a positive outcome, the initiative has to be easy* (AB-R interview).

Yellow Ribbon’s message is straightforward, and its task is simple. Besides, the colour yellow is symbolic and captivates people’s attention. Some of the neighbourhood associations which had not been involved in community development wanted to join the Yellow Ribbon drill. The later online survey showed 68.8% had seen a ribbon being put up in other households. Some said they had gone home to tie the ribbon outside (A-L interview). Such behaviour suggests participation can be contagious when tasks are made easy.

Yellow Ribbon was ‘a turning point’ for the Aruku Project:

*More than 60% participated, which is a very good result. I felt this was OK. It was proof of people’s awareness going up. It also was an opportunity for Aruku members to be reassured that they were doing right* (AB-R interview).

The Yellow Ribbon activity demonstrated a mutual effect, that is, one’s participation influences others’ participation. Many said, ‘I will evacuate next time’ (A-L interview). Having discovered that easy-to-partake initiatives can be contagious, the Aruku Project intends to make the most of this to increase the number of participants in Kawabe in preparing for future disasters.

### Satsuki Project in the Yata Ward, Mabi Town, Kurashiki City, Okayama Prefecture

#### Background and aims

**Figure 5:**
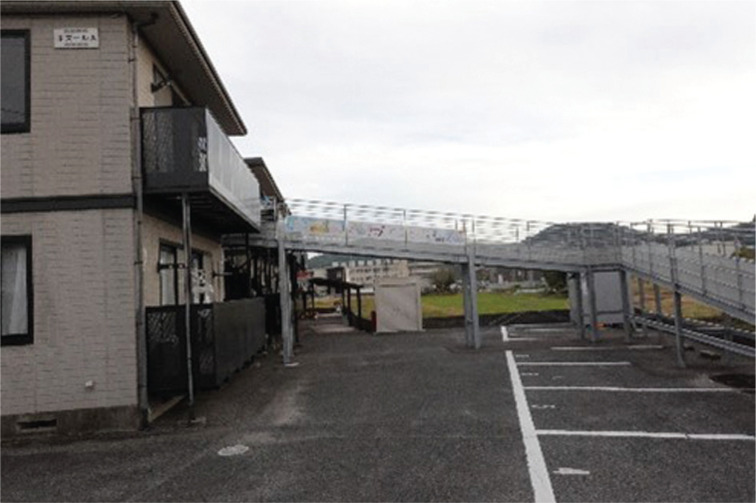
Satsuki Apart.

The Satsuki Project aims to build an inclusive cooperative community with an introduction of a new residential block that entails evacuation functions in the Yata Ward. The block has a nickname ‘Satsuki Apart’ (Fig. 5) – ‘satsuki’ means azalea, the flower emblem of Mabi Town. The project is run by Team Satsuki, which is a loosely-formed interest group comprising the founder of a local nursing home, a voluntary organisation for community development, a DRR researcher, the Social Welfare Council (Kurashiki City Hall), an NGO for disaster support and many more. The project derived from the issue of non-evacuation by the elderly and people with disabilities. Out of the 51 fatalities in the 2018 disaster in Mabi Town, 22 persons lost their lives on the ground floor having not being able to evacuate vertically despite living in two-storey buildings. The project identified three lessons learned from the disaster experience: a) ‘a disaster can occur any time. We need to prepare now for our super ageing society’; b) ‘preparedness of both infrastructure and lifestyle are important’; c) ‘disaster survivors are also co-creators of the community having something to offer’. To manifest these lessons, the project turned an inundated two-storey apartment block (previously flooded in the 2018 event), which was donated by its owner, into an innovative residential block adding evacuation functions. One function is the external long slope which enables wheelchairs or mobile beds to reach the upper-floor shelter space easily in a time of flooding. The shelter space is also a communal room, which is for social use for the block’s residents and also for the building’s neighbours. The residents also decided to store stockpiles and emergency kits in the room [52].

Once the building was completed and some survivors of the disaster moved in to rebuild their lives, the Satsuki Project shifted its focus to strengthening social connections, which would enhance community preparedness. Satsuki Apart’s residents, some of whom have difficulties living on their own, discussed how to develop a support mechanism in the block. The emphasis was also put on developing relationships beyond the facility with the neighbourhood as Satsuki Apart’s residents were not necessarily from the area. Yata Ward’s Machizukuri Committee has been involved in designing and implementing collaborative activities with the ward’s wider organisations and residents. The committee leader explains,

*it is important to develop links between Satsuki Apart and the neighbourhood; it is also important to know ‘the other’, to see their faces* (B-M interview).

For example, using the communal space in Satsuki Apart, the committee has arranged gatherings for singing to which the residents and neighbours are invited. A potato digging event in which Satsuki Apart’s residents harvested potatoes with the children from a nearby nursery was organised. According to the committee leader, Yata Ward’s *machizukuri* includes the principles of welfare and ‘warm’ community-building, as well as DRR, which match with Satsuki Project’s mission (B-M interview). Building positive intergenerational relationships has become a critical aspect of the project.

#### Satsuki Apart – ‘Social’

In the case of the Satsuki Project, its strategy for widening participation is to make an evacuation action usual and social. As the leader of the Satsuki Project says:

*evacuation requires courage. People have a lot of hesitation* (B-L interview).

She gives a list of examples: one of the Satsuki Apart’s residents was to stay at home because he could not move his bedridden wife; a family with an elderly person with memory loss did not want to go to a designated evacuation centre; a paralysed woman hid from the helicopter rescue. All of them did not wish to bother others.

*But people can evacuate to a familiar place* (B-L interview).

The more often the Satsuki Apart’s residents and neighbours walk to the communal room on the upper floor, the more confident they become of this action, that is, evacuation. Gathering at the Satsuki Apart for social and leisure purposes and joint local events allows them to get to know each other (B-L interview) [52]. Regularity and familiarity reduce psychological and physical barriers. There was an opportunity to test the objectives of the Satsuki Project during another heavy rain of 2020:

*residents and neighbours evacuated to here [the communal room], had a chat and a cup of tea, and nothing happened. They went home saying, ‘It was fun!’. It won’t happen if they haven’t been here before and didn’t know each other* (B-L interview).

At the Satsuki Apart, evacuation is no longer a difficult exceptional matter but a daily act of getting together with friends and neighbours who enjoy socialising.

### Evacuation Card Project in Miyoshi Ward, Ozu City, Ehime Prefecture

#### Background and aims

**Figure 6:**
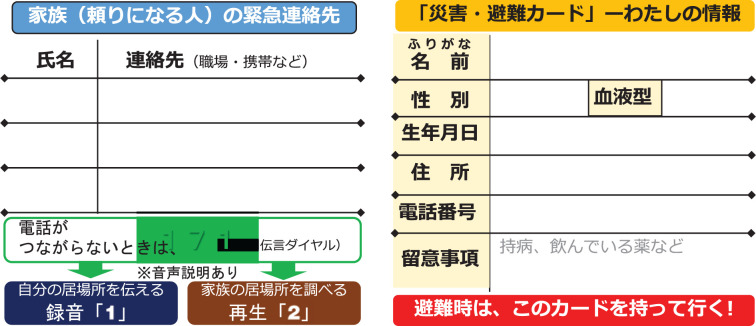
Disaster/Evacuation Card [53]. © 2017 Disaster Management, Cabinet Office.

The Miyoshi Ward had already been working on preparedness prior to the 2018 Heavy Rain Event, which makes their case different from others in terms of the timing of the learning from the previous disasters. Historically, Miyoshi Ward has faced inundation of water, which led to ‘bitter experiences’ (C-L interview) in the area. The 1943 disaster washed away all the houses, devastated agricultural produce and killed a firefighter. Protecting residents and agriculture is a matter of survival for the people of Miyoshi. In 2006, a voluntary disaster prevention organisation [*jishubosaisoshiki*] was set up in the Miyoshi Ward as the first case in Ozu City. The association then led the creation of the disaster prevention management plan completed in 2015. Their proactive attitude in DRR was also demonstrated in their application for the Cabinet Office’s Disaster/Evacuation Card Model Projects in 2016 (Fig. 6) [54]. Miyoshi Ward selected and developed two types of evacuation cards – a business card type and a leaflet type – through three community workshops. Eight groups were formed according to the possibilities of inundation of water identified from the 1943 event to discuss evacuation actions in the workshops – the community was learning from the previous disaster experience, even though it was more than a half-century ago. In the third workshop, using the evacuation cards, the groups took part in an evacuation drill and revised the contents of the cards afterwards. To disseminate the evacuation cards to every resident in the Miyoshi Ward, the neighbourhood association and the voluntary disaster prevention organisation requested 17 sub-wards to hold another workshop separately in 2017. The detailed information such as your temporary shelter and whom you will call out was filled into everyone’s card, which was put into a strapped plastic case to be hung, for example, on the fridge at home to make it visible at all times (C-L interview).

Miyoshi community’s efforts to learn from the bitter experience bore fruit. They were able to achieve no fatalities in the Heavy Rain Event of 2018, even though it was the largest disaster in the post-war era. When the torrential rain began, the members of the voluntary disaster prevention organisation set up a countermeasure headquarters at the public hall [*kominkan*] which was one of the designated evacuation shelters. They collected the information on the rainfall and the dam situation and also called for residents to evacuate, using local cable broadcasting, which prompted residents to leave. The headquarter later assessed the risk of the public hall being flooded and the timing of the secondary evacuation. The evacuees all moved to a substation facility on higher ground. The public hall was later inundated with flood water. During these evacuation processes, the Evacuation Cards were deemed effective. Residents were able to collect those whom they were supposed to help, which had been decided in the workshops and noted in the cards [21]. Owing to the Evacuation Card Project, ‘every resident has been able to learn the importance of protecting one’s life by oneself’ [54]. ‘We have a high awareness of water disasters and a strong motivation to protect ourselves against them’ (C-L interview).

#### Evacuation Card Project – ‘social’

Preparedness action in Miyoshi was undertaken under the leadership of the chairman of the neighbourhood association and the president of the voluntary disaster prevention organisation (C-M1 interview). The development of the disaster management plan or the Evacuation Cards would not have happened without their leadership. However, the leaders were aware that forcing participation would not make any impact. As the chairman said, ‘the plan wasn’t created only by certain seniors or executives in Miyoshi’ (C-L interview). Using cable broadcasting, the leadership team invited residents to join the process using the following persuasion:

*For a water disaster, we know it is coming, unlike an earthquake or tsunami. Our communities know the best what is happening at the time of a water disaster, authorities don’t. Circumstances differ from community to community. We cannot rely on kojo [public help]* (C-L interview).

Between 80 and 100 residents did gather (C-L interview). As a small rural community, ‘good relationships were already there’ (C-M1 interview) in the Miyoshi Ward. This high degree of social capital contributed to bringing the community together in the Evacuation Cards Project.

*We invited everyone to the workshops, those who were interested attended them and many of us studied together. This means there is always someone in the neighbourhood who is familiar with DRR* (C-L interview).

Even when some residents showed reluctance in engaging in the project, they were not pressurised. The leaders had a friendly conversation with them explaining ‘we have to do this for survival’ and encouraging them to participate (C-L interview). Those who were not keen at the beginning became the ones who appreciated the results the most after going through the flood when the cards saved them. Miyoshi’s widening participation strategy is by encouragement and invitation because of the established social relationships in the community.

### Nomura Recovery Project in Nomura Town, Seiyo City, Ehime Prefecture

#### Background and aims

Together with the other four towns, Nomura Town has been guided by Seiyo City Hall in the recovery processes after the 2018 disaster. Nomura had the largest damage in the city – five out of six fatalities, and 919 out of 1367 damaged households, including those fully destroyed [55]. The population in the town did not have a DRR awareness before the disaster (D-LO interview) and therefore had relied on the authority’s leadership. Despite such a top-down formation, the city hall’s intent was collaborative from the onset, presenting three principles in the city’s recovery *machizukuri* plan issued in March 2019: let us take time to recover from worry and grief by ‘standing next to each other and supporting one another’; recovery is about ‘one step of 100 persons, rather than 100 steps of one person’; all stakeholders including the public administration need ‘to prioritise carefully what has to be done’ [55]. Based on the city’s plan, Nomura Town developed its breakdown plan in October the same year. While outlining infrastructural recovery schemes, the October plan contains preparedness measures linked to the recovery schemes. For example, restoring the inundated riverbed of the Hiji River is an engineering project, but also an educational effort to prepare future generations for disasters by involving them in deciding on sustainable uses of the riverbed space.

#### Citizenship Education Project – ‘attractive’ and ‘social’

**Figure 7:**
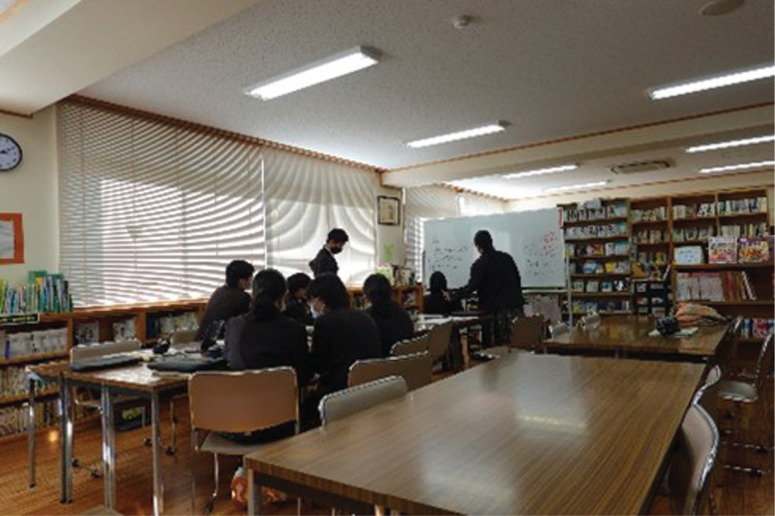
Investigatory Hour at Nomura High School.

This paper treats two initiatives that aim to foster public responsibility as well as disaster awareness in Nomura’s next generations as one Citizenship Education Project, although they derive from different objectives in the town’s plan. One emerged through the infrastructural *machizukuri* strand, which discusses the utilisation of the spacious riverbed of the Hiji River. After a series of workshops, the people of Nomura decided to develop vegetable, fruit and flower farms, a public park and sports fields utilising the space. In the discussion process, the researcher whose expertise is environmental engineering came up with an idea to involve Nomura High School (Fig. 7). Its Investigatory Hour [*Tankyuu no jikan*], which is a field-based, active-learning lesson, has been allocated for the planning and implementation of a project. A group of students chose to lead the management and maintenance of the farms (19/11/21 observation; D-R1 interview). When we observed their class at the high school, students were creatively discussing the dishes using the harvested sweet potatoes, which were to be served at the approaching major festival in the town (19/11/21 observation).

The other initiative is part of the DRR education package lessons for primary schools called ‘Learning From the Disaster’ coordinated by Seiyo City [56]. We observed a lesson delivered at Nomura Primary School, which uses its Integrated Study hour for DRR education (19/11/21 observation). A citizenship education expert from a university is invited to give lessons on preparedness education with a focus on fostering participation and citizenship. The primary school lesson had group discussions about the school’s stockpile storage – what to write on the note which will be displayed on the storage to inform evacuees what to do. An official from the city hall was there to offer feedback to students and also to borrow some of their ideas for implementation. Such procedures had been discussed between the school, the city hall and the university with a belief that students also have a stake in society and they should understand that [57].

Both educational activities at the high school and the primary school share a common theme – ‘becoming a citizen through social participation’ (D-R2 interview). It can be suggested the Nomura’s Citizenship Education Project allows for both fostering citizenship and widening participation through students’ social engagement in community initiatives. Starting early by targeting students is often regarded as an effective approach to cultivating DRR readiness [58]. The high school initiative also has some elements of attractiveness by collaborating with the local festival, which is the largest event in the town. This could motivate students to take part in, for example, the farm initiative. It is intended that students’ social action will be reinforced by receiving an affirmation from adults – the city hall taking their ideas on board, and customers paying for self-made dishes and enjoying them at the festival. These positive experiences encourage the learners to further engage in social action [57].

#### Capacity of the EAST framework

Table 5 summarises the widening participation strategy of each RPP. We were able to illuminate different approaches taken in four cases. Yellow Ribbon is a one-off and captivating initiative, whereas the other three are longer-term developmental initiatives that require social relationships. Within those three, Satsuki Apart and the Citizenship Education Project aim to build connections through social activities, while the Evacuation Card Project capitalises on the existing social capital to enable the project. One proposition here is that the EAST framework has the potential to guide a variety of participatory preparedness initiatives in terms of scale, target and objectives.

**Table 5.** Summary of the widening participation strategies of the four projects

Location	Kawabe Ward, Mabi Town, Kurashiki City	Yata Ward, Mabi Town, Kurashiki City	Miyoshi Ward, Ozu City	Nomura Town, Iyo City
**Project title**	Aruku Project	Satsuki Project	Evacuation Card Project	Nomura Recovery Project
**Disaster experience**	Historically had floods	Historically had floods	Historically had floods	Little
**Project aim**	Recovery, preparedness, machizukuri	Recovery, preparedness, machizukuri	Preparedness	Recovery, preparedness, machizukuri, citizenship
**Key initiative**	Yellow Ribbon	Satsuki Apart	Evacuation Card Project	Citizenship Education Project
**Strategy for widening participation**	Simple and clear task which has contagious effect	Socialising opportunities in daily life	Capitalising on strong social capital	Educating future generations
**After the disaster when people are most receptive (except Evacuation Card Project)**								
EAST*	E, A, T	S, T	S, T	A, S, T

*EAST: Easy, Attractive, Social, Timely.

## Strengthening the EAST framework

In considering the way forward of the EAST framework in the DRR context, this section attempts to link the four principles with the broad observations surfaced in the empirical study of the four RPPs beyond the key initiatives examined above. ‘E’ for making the action easier is akin to the idea of ‘lowering the hurdle’ commented on by the Aruku Project and the Satsuki Project (A-L interview; B-L interview). The idea was advocated by Yamori [59], who used generic language for better communication with the public. For many, the hurdle of participating in DRR activities is high. The reasons for this have been studied. One obvious response is a lack of time and resources. The denial that ‘a disaster will not happen to/affect me’ –‘the normalcy bias’ [60] – is another. People show reluctance or resistance when they perceive they are compelled to participate [61]. Lowering the hurdle is about making it accessible, approachable and embedded in daily life. The Yellow Ribbon activity conveys a clear message of safety through the simple act of hanging the ribbon at the door. Taking a small concrete step as such, rather than embarking on a grand design of a community-based plan, encourages people to get involved.

‘A’ for creating an attractive initiative can be translated into making it enjoyable. All RPPs, except the Evacuation Card Project, mentioned this principle was important. The Aruku Project, for example, was set up to create ‘something to look forward to’ (A-L interview) after the devastating disaster. People queue for the food bank but also arrive early to have a conversation while waiting, which gave them a moment of joy. Singing sessions in the Satsuki Project and joining with the local festival in the Citizenship Education Project share a similar mission to make their initiatives enjoyable to attract more participants. What we also came across in the interviews is the current participants (A-M1 interview; B-M interview) and also the researchers said, ‘I am having fun’ (AB-R interview; D-R1 interview) engaging in the projects. The fact that the participants are enjoying the projects may trigger others to participate.

‘S’ for emphasising social connections is probably the most frequently addressed principle in the RPPs we studied. The first example is the phrase ‘to be connected’ [*tsunagaru*], which was repeatedly used in the interviews. ‘Creating a space’ [*ba o tsukuru*] is an associated phrase, which refers to a means to allow people to be connected. The larger the number of familiar places or places to visit people have, the better their health and safety will be. Such an attempt was illustrated at Satsuki Apart with the social gathering space. When a disaster occurs, ‘calling out’ [*koe kake*] each other becomes critical to support the vulnerable and identify who is silent and therefore needs help. In Miyoshi Ward’s Evacuation Card Project, the residents decided who should call out to whom in an emergency. In the 2018 flooding, following the plan, they called out the ones they were supposed to call out to (C-M4 interview) resulting in no victims. For the response and recovery phase, ‘standing by side’ [*yorisou*] is used as a manner to support those who are affected by a disaster. ‘*Yorisou*’ literally means ‘to snuggle’ but has been used in the context of health and welfare and increasing in DRR ‘to be there’, not physically but psychologically. All of these phrases regularly appear in the policy domain, academia and media, forming a powerful social discourse stressing the ‘people first’ [62] approach in DRR and *machizukuri*.

The interviewees did not explicitly refer to ‘T’ for grabbing the momentum. However, RPPs’ decisions were consciously made to act on developing preparedness shifting from recovery efforts. For the Satsuki Project, it was when the apartment was built, and residents moved in. They began discussing concrete ideas for preparedness programmes. The shift for the Citizenship Education Project was when the breakdown plan specifically for Nomura was created. The timing is different in Miyoshi’s case, which embarked on the Evacuation Card Project between the past experience and a future possibility. Their momentum was not shortly after being hit by a disaster but during the usual time when they saw an opportunity to act on preparedness through the government scheme. Their case is a demonstration of the significance of preparedness action at a time when things are normal and no disaster is occurring.

Although the criteria of the framework were broadly applied at this stage, the above discussion demonstrates that the four cases of the existing RPPs entail aspects of EAST. This may make sense because they are recognised as good practices. Follow-up studies on whether these strategies or approaches have increased the number of participants as well as whether a strategy which combines all four principles has a better outcome than a strategy based on one principle are required. With more empirical data, the EAST framework can be consolidated as a compatible frame of reference which guides communities to develop effective plans.

## Conclusion

This paper has argued that the perspectives on widening participation are significant in DRR contexts given there are many ongoing participatory and community-based projects that share recruitment challenges. One limitation seems to remain. There is a discrepancy between widening participation as a government policy and widening participation as a community initiative, which needs to be taken into account. The difference may manifest in methodology. Behavioural insights research utilises ‘rigorous testing and trialling based on a rich understanding of the context in which a policy is being delivered’ [49]. Communities are likely to have rich understandings of their contexts but unlikely to have resources for ‘rigorous testing and trialling’. How to enhance rigour in studying widening participation in community projects requires further investigation. Despite this, we suggest the EAST framework could address a new dimension of participation which has been seen in neither success factors frameworks nor participation typologies. Together with the existing theories, the EAST framework would be beneficial in co-creation and democratic decision-making.

## Data Availability

The data that support the findings of this study are available from the research participants but restrictions apply to the availability of these data, which were used under licence for the current study, and so are not publicly available. Data are however available from the authors upon reasonable request and with permission of the research participants.
